# Contractile effects of dexmedetomidine in the human heart

**DOI:** 10.1007/s00210-025-04192-x

**Published:** 2025-05-29

**Authors:** Joachim Neumann, Uwe Kirchhefer, Britt Hofmann, Ulrich Gergs

**Affiliations:** 1 Institute for Pharmacology and Toxicology, Medical Faculty, Martin-Luther-University Halle-Wittenberg, Magdeburger Str. 4, Halle (Saale), D- 06097 Germany; 2 Institute for Pharmacology and Toxicology, Medical Faculty, Domagkstr. 12, University Münster, Münster, D- 48149 Germany; 3 Department of Cardiac Surgery, Mid-German Heart Centre, University Hospital Halle, Ernst Grube Str 40, Halle (Saale), D- 06097 Germany

**Keywords:** Dexmedetomidine, H_2_-histamine-receptors, Transgenic mice, Human atrium

## Abstract

Dexmedetomidine is an approved drug that is chemically related to clonidine. Dexmedetomidine is used to induce sedation and anxiolysis. These therapeutic effects of dexmedetomidine are explained by its agonistic action on brain α_2_-adrenoceptors. We tested the hypothesis that dexmedetomidine like clonidine also stimulated human cardiac atrial H_2_-histamine-receptors. We noted that 10 µM dexmedetomidine increased force of contraction in electrically stimulated (1 Hz) human right atrial preparations (HAP, obtained during open heart surgery). These effects were increased by previously applied cilostamide and slightly attenuated by subsequently applied 10 µM cimetidine but greatly attenuated by 10 µM propranolol or when first 10 µM cocaine was given. After pre-stimulation with histamine, subsequently applied 1 µM dexmedetomidine reduced force of contraction in HAP. In left atrial preparations from mice with cardiac-specific overexpression of H_2_-histamine receptors (H_2_-TG), we noted a positive inotropic effect of 10 µM dexmedetomidine in the presence of 100 nM rolipram that was reversed by cimetidine. In left atrial preparations of wild-type mice, 10 µM dexmedetomidine increased force of contraction in the presence of rolipram which was reversed by subsequently applied 10 µM propranolol and attenuated by pretreatment with 10 µM cocaine. These data indicate a direct cardiac action of dexmedetomidine. Dexmedetomidine can probably, in principle, release endogenous cardiac noradrenaline. Thus, dexmedetomidine can act as a partial agonist at human cardiac H_2_-histamine receptors but its main effect in HAP is the release of endogenous β-adrenergic catecholamines.

## Introduction

**Fig. 1:**
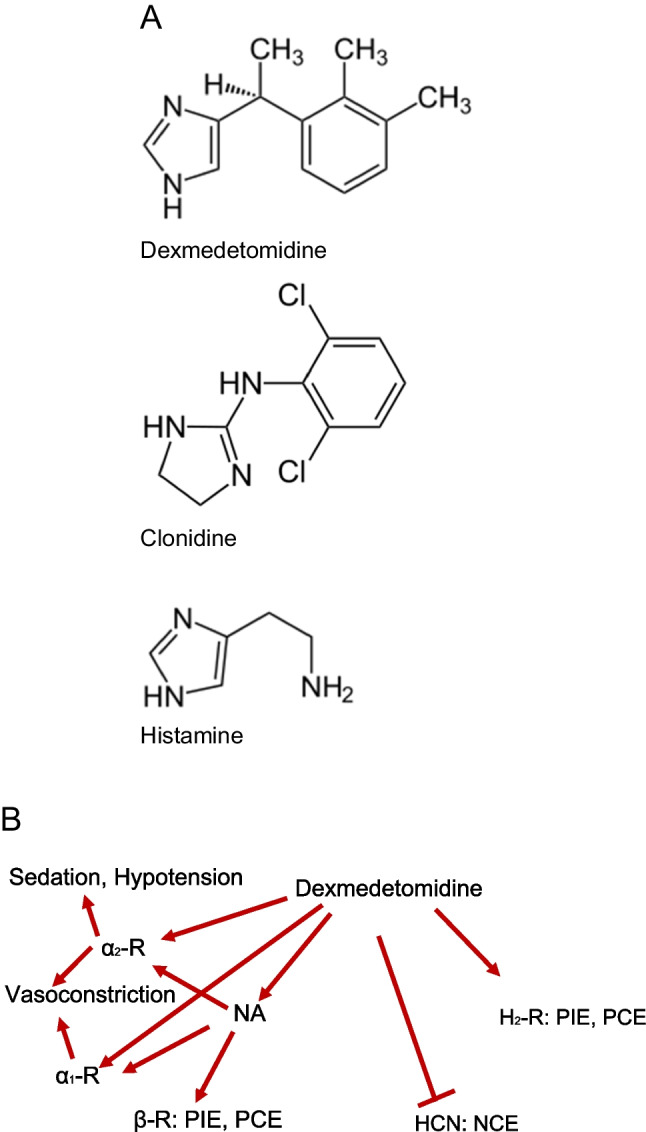
**A** Comparison of the structural formulae of dexmedetomidine, clonidine, and histamine. Consider that dexmedetomidine contains a chiral carbon atom. Please note that clonidine and dexmedetomidine share similarities in their heterocycles but also show discrepancies in their structures. This might explain why dexmedetomidine and clonidine have overlapping but not identical receptor binding profiles. **B** Dexmedetomidine might directly stimulate α_1_-adrenoceptors, α_2_-adrenoceptors, or H_2_-histamine receptors to induce positive inotropic effects and positive chronotropic effects, or may stimulate HCN to induce negative chronotropic effects, may release noradrenaline (NA) which may stimulate β-adrenoceptors (β-R), and then could cause positive inotropic effect and positive chronotropic effects

Dexmedetomidine is chemically related to clonidine (Fig. 1A, Virtanen et al. 1988). The clinical usages of clonidine and dexmedetomidine overlap (Masuki et al. 1985, Weerink et al. 2017). Dexmedetomidine is employed in the intensive care unit to bring about sedation or anxiolysis and to reduce the need for analgetic drugs after surgery in ventilated patients (Weerink et al. 2017). Dexmedetomidine acts probably as a hypnotic anesthetic drug via stimulation of brain α_2_-adrenoceptors (Schwinn et al. 1991, Yildiz et al. 2007). These α_2_-adrenoceptors couple through pertussis-toxin-sensitive guanosine triphosphate (GTP)-binding-proteins (Doze et al. 1990, Hamasaki et al. 2002). Dexmedetomidine is more potent and selective than clonidine at α_2_-adrenoceptors (Weerink et al. 2017).

Dexmedetomidine was developed from medetomidine. Dexmedetomidine is the active D-isomer of medetomidine (4-[1-(2,3-dimethylphenyl)ethyl]− 1H-imidazole). Dexmedetomidine was invented by the Finnish drug company Orion® (Virtanen et al. 1988, Savola and Virtanen 1991). Based on ligand binding or functional studies, medetomidine was reported to lack effects on β_1_-, β_2_-, H_1_-, 5-HT_1_-, 5-HT_2_-, muscarinic, dopamine-, tryptamine-, GABA-, opiate-, and benzodiazepine-receptors (Virtanen et al. 1988). Hence, we might assume that also dexmedetomidine is ineffective at these receptors. Racemic medetomidine is employed in veterinary medicine (Fig. 1A, Virtanen et al. 1988, Weerink et al. 2017). Up to 3 µM medetomidine failed to alter basal force of contraction or basal spontaneous beating rate in isolated guinea-pig left or right atrial preparations (Virtanen et al. 1988). Now, histamine alone is known to induce a positive chronotropic effect via H_2_-histamine receptors in guinea-pig right atrial preparations (review: Neumann et al. 2021a). However, when histamine had increased beating rate in guinea-pig right atrial preparations, then 30 µM medetomidine failed to reduce the beating rate (Virtanen et al. 1988). They drew the conclusion that and therefore dexmedetomidine failed to stimulate or inhibit H_2_-histamine receptors, at least in the guinea pig heart. There is functional evidence that at least medetomidine (30 µM) did not reduce the positive chronotropic effect of isoprenaline or histamine in right atrial guinea-pig preparations (Virtanen et al. 1988). Thence, medetomidine had no functional anti-β-adrenergic or anti-H_2_-histaminergic effects in guinea-pig atrial preparations (Virtanen et al. 1988). In isolated papillary muscles from reserpinized ferrets, dexmedetomidine at 10 µM had no positive inotropic effect, and in these muscles, dexmedetomidine did not increase Ca^2+^-transients (Housmans 1990). However, 10 µM dexmedetomidine raised the maximum rate of tension relaxation in isolated papillary muscles from these reserpinized ferrets (Housmans 1990). The underlying mechanism why dexmedetomidine raised the rate of muscle relaxation in isolated papillary muscles from reserpinized ferrets was not reported (Housmans 1990). Clonidine is an agonist at human H_2_-histamine receptor in H_2_-TG but more importantly in HAP (Neumann et al. 2023b). Dexmedetomidine contains an imidazole ring like histamine (Fig. 1A). Hence, from this chemical similarity, one might think that dexmedetomidine might bind to histamine receptors. Others failed to find any effect of dexmedetomidine alone or any effect of dexmedetomidine on the histamine-stimulated beating rate in guinea pig right atrial cardiac preparations (Virtanen et al. 1988). This positive chronotropic effect of histamine in the guinea pig heart is known to be H_2_-histamine receptor-mediated and thus the authors ruled out both an agonistic and antagonistic action of dexmedetomidine on H_2_-histamine receptors in the guinea pig heart (Virtanen et al. 1988).

Here, we also wanted to compare our prior findings on clonidine with those with dexmedetomidine. Parts of this work were published in an abstract form (Neumann et al. 2024).

Thus, we tested mainly the hypotheses that:

1. dexmedetomidine increased force of contraction in left atrial preparations of H_2_-TG via H_2_-histamine receptors.
2. dexmedetomidine increased beating rate in right atrial preparations of H_2_-TG via H_2_-histamine receptors.
3. dexmedetomidine increased force of contraction in HAP via H_2_-histamine receptors.
4. dexmedetomidine increased force of contraction in HAP and left atrial preparations via release of noradrenaline.

## Materials and methods

### Transgenic mice

#### Contractile studies in mice

In brief, the right atrial preparations and left atrial preparations from wild-type mice and H_2_-TG (12 animals, random gender, about 200 days of age), generated as previously described, were isolated and mounted in organ baths as published (Gergs et al. 2013, Gergs et al. 2019, Neumann et al. 1998). The bathing solution of the organ baths contained a modified Tyrode’s solution comprised of 119.8 mM NaCI, 5.4 mM KCI, 1.8 mM CaCl_2_, 1.05 mM MgCl_2_, 0.42 mM NaH_2_PO_4_, 22.6 mM NaHCO_3_, 0.05 mM Na_2_EDTA, 0.28 mM ascorbic acid, and 5.05 mM glucose. Ascorbic acid was used to inhibit oxidation of drugs like histamine. The solution was continuously gassed with 95% O_2_ and 5% CO_2_ and maintained at 37 °C and pH 7.4 (Neumann et al. 2021a, b). Left atrial preparations were electrically stimulated at 1 Hz with a voltage of 3–4 V, with a duration of 5 ms. Left atrial preparations were used to study force of contraction and other parameters of muscle contraction as time of contraction or the first derivative of force versus time. Spontaneously beating right atrial preparations from mice were used to study any chronotropic effects. Force was measured under isometric condition with use of force transducer (Hellige, Freiburg, Germany) a bridge amplifier (ADInstruments, Oxord, England) and signals were electronically assessed with a dedicated software (Labchart 8 also from ADInstruments) on a personal computer. Drug application was as follows. After equilibration was reached, drugs were cumulatively added to left atrial or right atrial preparations to establish concentration-response curves. Then, where indicated, cimetidine and propranolol subsequently were added to the preparations. In some studies, first 10 µM cocaine was given. For details, see figure legends and original tracings. On each experimental day, we studied usually one wild-type mouse and one transgenic mouse at the same time in four organ baths which are identically designed and stood in line. They contained alternatively left atria and right atria. We were pipetting the solutions at the same time into these organ baths. Hence, any diffusional limits (e.g., for oxygen in such multicellular preparations) would be present both in transgenic and wild-type atria.

#### Contractile studies on human preparations

In brief, human atrial preparations were obtained during cardiac surgery at the sites where needles for extracorporeal circulation were cut into the right atrial appendages. These samples were rapidly (in about half an hour) transferred into the pharmacological laboratory in beakers containing the modified Tyrode’s solution described in the preceding section. The contractile studies on HAP were performed using the same modified Tyrode’s solution and organ baths and stimulators as for mouse atria. Muscle strips (HAP) were stretched to optimal length that allowed maximal generation of force of contraction. HAP were stimulated (1 Hz) electrically with platinum electrodes with rectangular impulses of direct currents from a Grass stimulator SD 9 (Quincy, MA, USA). Voltage was set around 10 V, just sufficient to initiate contractions. Electrical impulses had a length of 5 ms. The signals from the force transducer (Hellige, Freiburg, Germany) went into a bridge amplifier (ADInstruments). Signals were digitized and stored on a commercial personal computer. The signals were quantified using a commercial software (Lab Chart 8 from ADInstruments bought through their distributor in Oxford, England). The muscle strips were then mounted vertically under isometric conditions with metal hooks at each end of the muscle in double barrelled glass organ baths. Thus, the contractile studies on human preparations were done using the same setup and buffer as used in the mouse studies (vide supra). The samples were from ten male and four female patients. Their age ranged between 64 and 82 years. The indication for bypass surgery was severe coronary heart disease (two- or three-vessel disease). Cardiac relevant comorbidities included atrial fibrillation, hypertension, heart failure, diabetes mellitus type 2, and overweight. Cardiac drug therapy included acetyl salicylic acid, apixaban or similar anti-coagulants, furosemide or similar diuretics, and bisoprolol or similar β-adrenoceptor antagonists. Patients gave written informed consent to participate in this study. Our methods used for atrial contraction studies in human samples have been previously published and were not altered in this study (Gergs et al. 2009, 2017). We usually tried to obtain two HAP from each patient which show similar contractile behavior. Samples are only considered if they developed under basal conditions at least 1 mN of force. Treatments are usually run in parallel in these HAP. For instance, one sample was treated for 10 min with 10 µM cocaine, then both HAP undergo a concentration response curve to dexmedetomidine. This was done to reduce scatter of data. However, the patients are operated upon because they have cardiac morbidities and hence differences in basal tension between patients are an unavoidable limitation of the studies that we are reporting here.

### Data analysis

Data shown are means ± standard error of the mean. Statistical significance was estimated using the analysis of variance followed by Bonferroni’s *t*-test or Student’s paired *t*-test as appropriate. A probability value < 0.05 was considered to be significant.

### Drugs and materials

The drugs dexmedetomidine (10 mM dissolved in dimethylsulfoxide (DMSO)), histamine dihydrochloride, rolipram, cilostamide, propranolol, and cocaine were purchased from Sigma-Aldrich (Taufkirchen, Germany) or Tocris/Bio-Techne (Wiesbaden, Germany). All other chemicals were of the highest purity grade commercially available. Deionized water was used throughout the experiments. Stock solutions were prepared fresh daily.

## Results

**Fig. 2:**
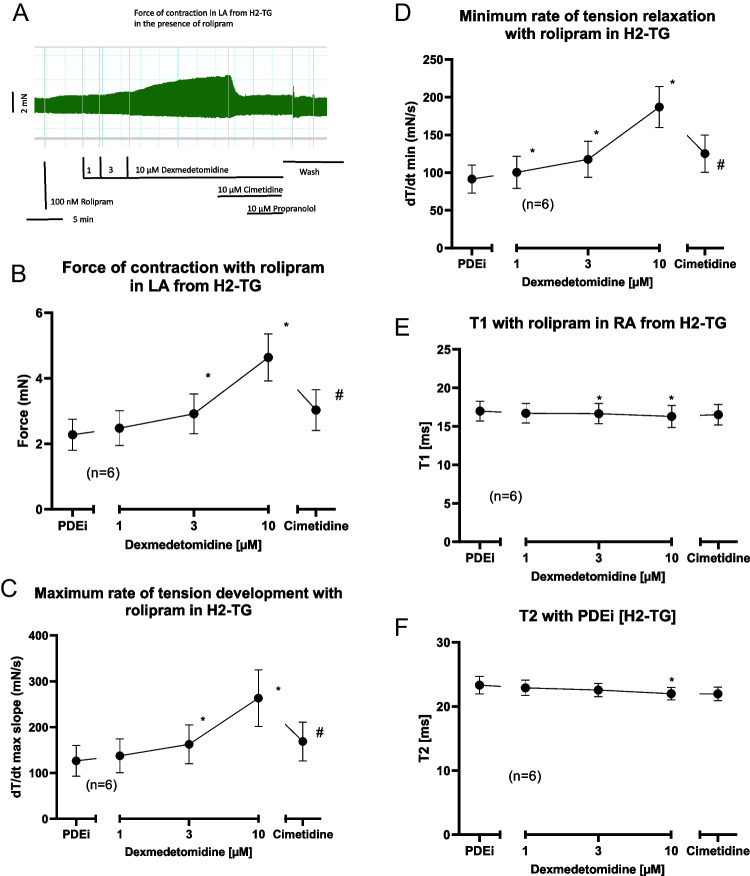
Dexmedetomidine induced a concentration-dependent positive inotropic effect in left atrial preparations from H2-TG. First 100 nM rolipram (PDEi) was applied. Then dexmedetomidine was cumulatively added and finally a maximum effective concentration of cimetidine (10 µM) and then propranolol (10 µM) was employed. Ordinates in Fig. 2**A**, 2**B**, 2**C**, 2**D**, 2**E** and 2**F** depict force of contraction in milli Newton (mN), maximum rate of tension development (+dF/dt), maximum rate of tension relaxation (-dF/dt), time to peak tension (T1 in milliseconds: ms) or time of relaxation (T2 in ms.), respectively. Abscissae depict concentrations of histamine in negative logarithmic molar units. * *p*< 0.05 vs. Ctr, # *p*< 0.05 vs. 10 µM dexmedetomidine. Number in brackets means number of experiments

**Fig. 3:**
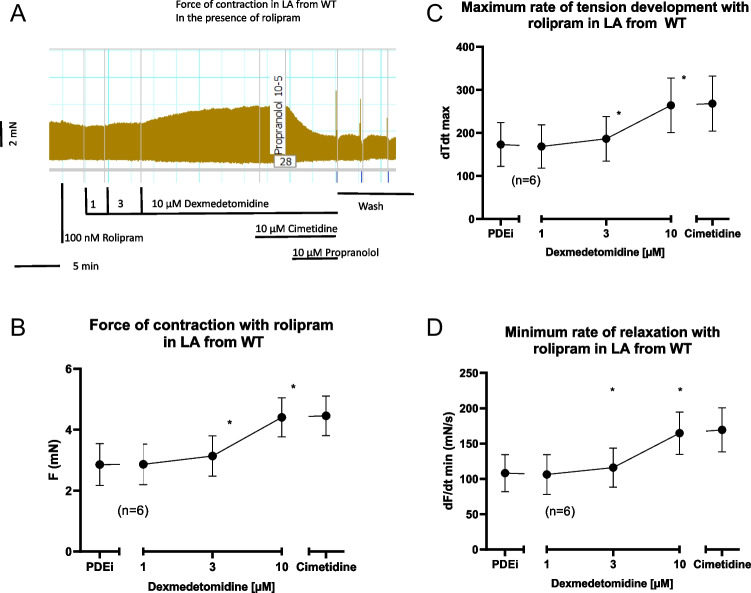
Dexmedetomidine induced a concentration-dependent positive inotropic effect in left atrial preparations from wild-type mice. First, 100 nM rolipram (PDEi) was applied. Then, dexmedetomidine was cumulatively added and finally a maximum effective concentration of cimetidine (10 µM) and then propranolol (10 µM) was employed. Ordinates in **A**, **B**, **C **and **D** depict force of contraction in milli Newton (mN), maximum rate of tension development (+dF/dt), and maximum rate of tension relaxation (-dF/dt), respectively. Abscissae depict concentrations of dexmedetomidine in negative logarithmic molar units. * *p*< 0.05 vs. Ctr, # *p*< 0.05 vs. 10 µM dexmedetomidine. Number in brackets means number of experiments

Dexmedetomidine (1–10 µM) per se exerted a small (about 10 %) concentration-dependent positive inotropic effect in left atrial preparations from H_2_-TG but not from wild-type mice. We used these concentrations of dexmedetomidine based on previous work (Housmans 1990). This effect was considered too small to be studied further (data not shown). We next tried to amplify this effect by a phosphodiesterase inhibitor as we did before (Neumann et al. 2023a). We used the phosphodiesterase IV inhibitor rolipram (100 nM) because in mouse heart phosphodiesterase IV is mainly relevant for the degradation of cAMP. In the presence of 100 nM rolipram, dexmedetomidine exerted a concentration-dependent positive inotropic effect in left atrial preparations from H_2_-TG. This effect was reconsidered large enough to merit further study. This is seen in an original recording (Fig. 2A) and summarized for several experiments in Fig. 2B. Likewise, dexmedetomidine increased the maximum rate of tension development (Fig. 2C) and the maximum rate of tension relaxation (Fig. 2D). This latter parameter was mainly calculated, because in ferret papillary muscles, dexmedetomidine alone had raised the rate of muscle relaxation without increasing force of contraction which is quite remarkable (Housmans 1990). As it is typical for cAMP-dependent contractile effects (Li et al. 2000), dexmedetomidine shortened time to peak tension (Fig. 2E) and time of relaxation (Fig. 2F). Hence, we applied the H_2_-antagonist cimetidine to find out whether this effect could be reversed (Fig. 2A). This was the case (Fig. 2A). Unexpectedly also in left atrial preparations from wild-type mice, dexmedetomidine raised force of contraction in the presence of rolipram. This is seen in an original recording (Fig. 3A). Data from several such contraction experiments are summarized in Fig. 3B. Likewise, dexmedetomidine increased the maximum rate of tension development (Fig. 3C) and the maximum rate of tension relaxation (Fig. 3D). However, this effect was not H_2_-histamine receptor mediated, because it was not antagonized by cimetidine (Fig. 3A) but by propranolol (Fig. 3A).

A different picture emerged in right atrial preparations. In right atrial preparations from H_2_-TG, dexmedetomidine failed to alter the beating rate alone or in the presence of 100 nM rolipram (data not shown). This was unexpected, because usually compounds that stimulate H_2_-histamine receptors have a positive chronotropic effect in right atrial preparations from H_2_-TG (Gergs et al. 2019). One might speculate that positive chronotropic effects of dexmedetomidine are overtaken here by negative chronotropic effects via other unknown receptors. This was not studied further.

**Fig. 4:**
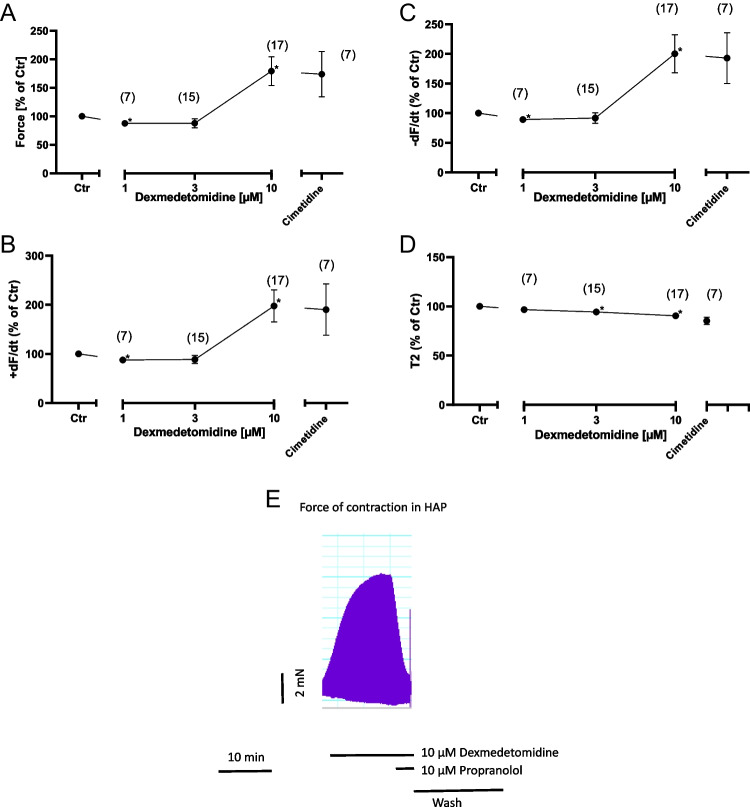
Dexmedetomidine induced a concentration-dependent positive inotropic effect in HAP. Dexmedetomidine was cumulatively added and finally a maximum effective concentration of cimetidine (10 µM) was employed. Ordinates in **A**, **B**, **C**, and **D** depict force of contraction in milli Newton (mN), maximum rate of tension development (+dF/dt), maximum rate of tension relaxation (-dF/dt), and time of relaxation (T2). Abscissae depict concentrations of dexmedetomidine in negative logarithmic molar units. * *p*< 0.05 vs. Ctr, # *p*< 0.05 vs. 10 µM dexmedetomidine. Numbers in brackets means number of experiments. **E** Original recording depicting a positive inotropic effect of 10 µM dexmedetomidine. Additional applied 10 µM propranolol rapidly and effectively reduced force of contraction. Then, the bath was washed by exchanging the buffer in the bath. Horizonal bar indicates time in minutes (min). Vertical bar indicates force of contraction in milli Newtons (mN)

Next, we wanted to know whether our findings in mice have any clinical relevance. Therefore, we incubated contracting HAP with dexmedetomidine. One micromolar dexmedetomidine reduced slightly force of contraction (Fig. 4A). However, 10 µM dexmedetomidine increased force of contraction (Fig. 4A). A similar pattern was noted on the maximum rate of tension development, on maximum rate of relaxation, and time of relaxation that are summarized in Fig. 4B, C and D.

An original tracing is seen in Fig. 4E; a single concentration of 10 µM dexmedetomidine greatly increased force of contraction, and this effect was rapidly and effectively reversed by propranolol (Fig. 4E).

**Fig. 5:**
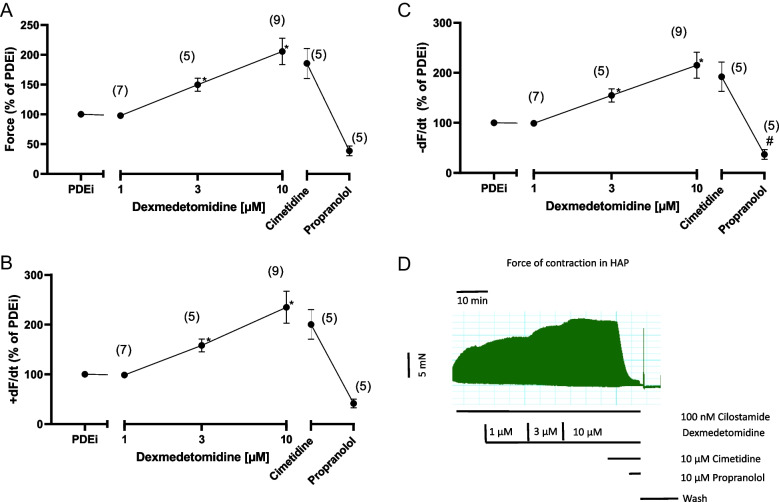
Dexmedetomidine induced a concentration-dependent positive inotropic effect in HAP. First, 100 nM cilostamide (PDEi) was applied. Then, dexmedetomidine was cumulatively added and finally a maximum effective concentration of cimetidine (10 µM) was employed. Ordinates in **A**, **B**, and **C** depict force of contraction in milli Newton (mN), maximum rate of tension development (+dF/dt), and maximum rate of tension relaxation (-dF/dt). Abscissae depict concentrations of dexmedetomidine in negative logarithmic molar units. * *p*< 0.05 vs. Ctr, # *p*< 0.05 vs. 10 µM dexmedetomidine. Numbers in brackets mean number of experiments. **D** Original recording depicting a positive inotropic effect of first cilostamide in HAP, then increasing concentrations of dexmedetomidine were cumulatively applied. Addition of firstly cimetidine and then propranolol reduced force of contraction. Then, the bath was washed by exchanging the buffer in the bath. Horizonal bar indicates time in minutes (min). Vertical bar indicates force of contraction in milli Newtons (mN)

Interestingly, the positive inotropic effect of dexmedetomidine was more pronounced in the presence of the phosphodiesterase inhibitor cilostamide (Fig. 5). We used in the HAP cilostamide, a phosphodiesterase III inhibitor, instead of rolipram, a phosphodiesterase IV inhibitor. This was simply done because we and others noted that cilostamide failed to increase force of contraction in mouse atrium and rolipram failed to increase force of contraction in human atrium (e.g., Neumann et al. 2023a, b). This observation is consistent with biochemical data that in the mouse heart phosphodiesterase IV and in the human heart phosphodiesterase III are predominant to degrade cAMP. Thus, in the presence of cilostamide, a positive inotropic effect to dexmedetomidine occurred already at 3 µM (Fig. 5A) and not only at 10 µM, as was the case in the absence of cilostamide (Fig. 4). We did not obtain a plateau in Fig. 5A and therefore it was not possible to calculate EC_50_-values for dexmedetomidine.

We note this pattern of an increase in contractile parameters not only for force of contraction (Fig. 5A), but also for the maxima of the rate of tension development (Fig. 5B) and the maxima of the rate of tension relaxation (Fig. 5C). Such data are also presented as original tracings: In the presence of cilostamide, increasing concentrations of dexmedetomidine increased force of contraction. While additional cimetidine was hardly effective, propranolol swiftly and efficiently reduced force of contraction (cf. Fig. 5D).

**Fig. 6:**
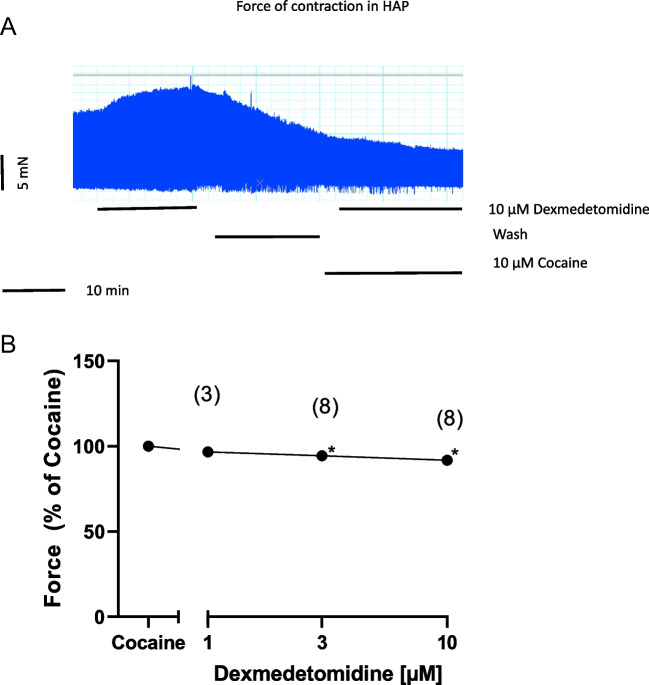
Cocaine impeded the positive inotropic effect of dexmedetomidine and induced a concentration-dependent negative inotropic effect in HAP. **A** Original recording depicting a positive inotropic effect if 10 µM dexmedetomidine in HAP. Then wash of the organ bath followed to reduce force of contraction. Thereafter, cocaine was given and later again 10 µM dexmedetomidine was used but then failed to increase force of contraction. Horizonal bar indicates time in minutes (min). Vertical bar indicates force of contraction in milli Newtons (mN). Ordinate in (**B**) depicts force of contraction in % of the effect from cocaine. Abscissa depicts concentrations of dexmedetomidine in negative logarithmic molar units. * *p*< 0.05 vs. Ctr. Number in brackets means number of experiments

From the literature, it was reported that dexmedetomidine did not bind to β-adrenoceptors (Virtanen et al. 1988). Thus, a direct sympathomimetic effect of dexmedetomidine was deemed unlikely from the start. To confirm or refute this view, as in previous studies (Neumann et al. 2023b), we first applied 10 µM cocaine and then gave dexmedetomidine in HAP. This is reported in an original tracing (Fig. 6A) and several experiments are summarized in Fig. 6B. Cocaine is assumed to block the entrance of drugs through transporters into cells most typically nerve cells in the HAP. Hence, dexmedetomidine might not enter cells and thus cannot release noradrenaline from cells. Thereby, noradrenaline cannot exert a positive inotropic effect in HAP via adrenoceptors. Indeed, in the presence of cocaine, dexmedetomidine failed to increase but even marginally decreased force of contraction in HAP (Fig. 6A and B). Hence, dexmedetomidine is in all likelihood acting as an indirect sympathomimetic agent in HAP.

**Fig. 7:**
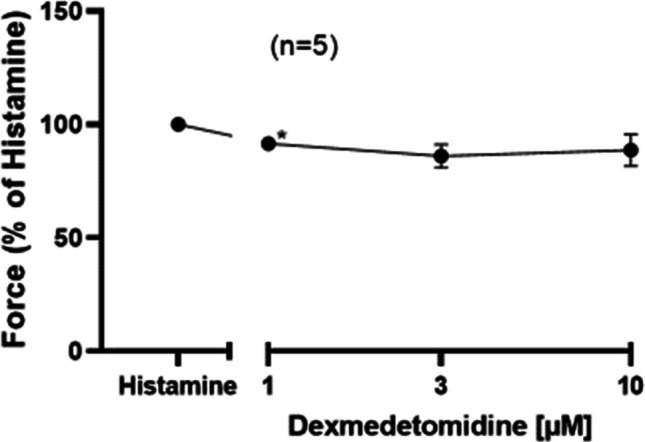
Histamine induced a positive inotropic effect in HAP. Then, dexmedetomidine was cumulatively added. Ordinate in Figure 7 depicts force of contraction % of histamine effect. The abscissa indicates dexmedetomidine in negative logarithmic molar units. * *p*< 0.05 vs. Ctr. Number in brackets means number of experiments

Now, one might envision that if dexmedetomidine is an ineffective agonist at H_2_-histamine receptors in the HAP, dexmedetomidine might be a potent and effective antagonist. To study this hypothesis, we first gave histamine to raise force of contraction in HAP and then we added dexmedetomidine. Under these conditions, 1 µM but not higher concentrations of dexmedetomidine reduced histamine stimulated force of contraction in HAP (Fig. 7).

**Fig. 8:**
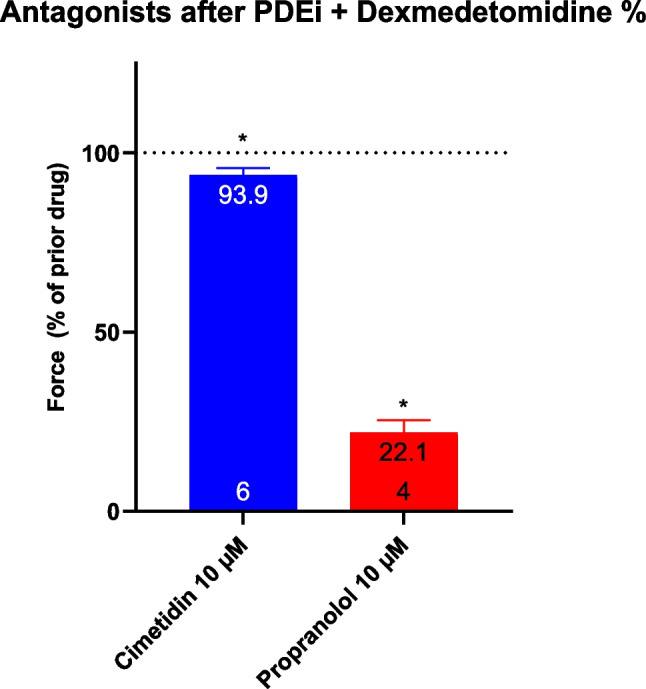
Effect of cimetidine or propranolol on the positive inotropic effect of dexmedetomidine in the presence of cilostamide in HAP. First, 100 nM cilostamide (PDEi) was applied (Ctr). Then, dexmedetomidine was cumulatively added (see Figure 5) and finally a maximum effective concentration of cimetidine (10 µM) or propranolol (10 µM) was employed. Ordinate in Figure 8 depicts force of contraction * *p*< 0.05 vs. Ctr. Number in bars means number of experiments

Finally, we have compared how the positive inotropic effect of dexmedetomidine in HAP in the presence of cilostamide could be attenuated by receptor antagonists. It turns out that only to a very small extent (less than 10 %) the positive inotropic effect of dexmedetomidine is weakened by cimetidine and to a major extent (more than 90 %) by propranolol (Fig. 8). Hence, we suggest that the positive inotropic effect of dexmedetomidine in man can hardly be explained by a stimulation of H_2_-histamine receptors.

## Discussion

The main new findings in this study are as follows: dexmedetomidine can increase force of contraction in HAP. This positive inotropic effect is probably due to a release of noradrenaline from cardiac stores.

### **Mechanism of dexmedetomidine**

We suggest that dexmedetomidine is, in large part, an indirect sympathomimetic agent in HAP. This is concluded from the following observations: dexmedetomidine increased force of contraction in the human atrium. This positive inotropic effect was blocked by propranolol, a β-adrenoceptor antagonist. Hence, the positive inotropic effect of dexmedetomidine in HAP must involve β-adrenoceptors. The positive inotropic effect of dexmedetomidine in HAP is blocked by cocaine; thence, positive inotropic effect involves an uptake process. Thus, it seems plausible that dexmedetomidine enters cells in the HAP that store noradrenaline and then it releases noradrenaline from, e.g., nerve cells. This released noradrenaline then stimulates β-adrenoceptors on cardiomyocytes and force increases. Theoretically, the cardiac action of dexmedetomidine might involve stimulation of α_2_-adrenoceptors in the heart. However, these receptors are usually thought to inhibit the activity of adenylyl cyclase which should lead to a negative inotropic and not a positive inotropic effect (Fig. 1B). Moreover, an effect via α_2_-adrenoceptors should not be sensitive to propranolol. Therefore, we think we must exclude α_2_-adrenoceptors as mediators for the positive inotropic effect of dexmedetomidine in HAP. Of note, our findings underscore that studies in mice even transgenic mice overexpressing human receptors can be very misleading. From our data in H_2_-TG, one would have predicted a similar positive inotropic effect of dexmedetomidine in HAP to be mediated by H_2_-histamine receptors. In contrast, to expectation, dexmedetomidine practically failed to raise force of contraction in HAP via H_2_-histamine receptors. Quite oppositely, dexmedetomidine reduced histamine-stimulated force of contraction in HAP (Fig. 7). This can be explained by an antagonistic action of dexmedetomidine at human cardiac H_2_-histamine receptors. Hence, we propose that dependent on the biochemical environment (mouse versus human heart) dexmedetomidine stimulates and/or inhibits H_2_-histamine receptors.

From our data in mice, it is clear that dexmedetomidine can stimulate human H_2_-histamine-receptor: firstly, dexmedetomidine only increased force of contraction in left atrial preparations from H_2_-TG and not from wild-type mice. Secondly, the positive inotropic effect of dexmedetomidine is reversed in left atrial preparations from H_2_-TG by cimetidine and not by propranolol (Fig. 2A).

When one compares dexmedetomidine with clonidine, one can delineate: in contrast to clonidine, dexmedetomidine increased force of contraction only in the presence of rolipram in left atrial preparations from wild-type mice. In contrast to clonidine alone, dexmedetomidine alone hardly increased force of contraction in H_2_-TG. Only in the presence of rolipram, dexmedetomidine increased force of contraction in H_2_-TG.

In HAP, clonidine increased force of contraction: an effect antagonized by both prazosin (to a minor part) and mainly by cimetidine (Neumann et al. 2023a). The positive inotropic effect of dexmedetomidine in HAP was only slightly attenuated by cimetidine and was thus to a minor extent mediated by H_2_-histamine receptor but mainly due to a release of endogenous noradrenaline. Hence, the pharmacology of dexmedetomidine is clearly different from that of clonidine in the human heart (Neumann et al. 2023a).

Clonidine causes sedation and provides analgesic relief and has been used in ventilated patients in intensive care. It was found that the usefulness of clonidine was impaired by its long half-life (6 to 10 h, Ebert et al. 2000, Shehabi et al. 2019, Wang et al. 2024). For that reason, similarly agonistic drugs at brain α_2_-adrenoceptors with a shorter half-life than clonidine were looked for and this led to dexmedetomidine. Dexmedetomidine has a half-life of 2 to 3 h and is eight- to tenfold more potent as an agonist at α_2_-adrenoceptors than clonidine (Ebert et al. 2000). In a previous report (Housmans 1990), dexmedetomidine failed to increase force of contraction in milli Newton and also when force was plotted as percentage of pre-drug value. However, if one carefully examines the original data, there is also an increase in mean values of force when concentrations of dexmedetomidine were raised. However, the scatter of data was high and perhaps therefore no significance was reached. Indeed, the errors bars in rate of relaxation were lower than in other force parameters and this may explain why in this single parameter a significant effect was reported (Housmans 1990). Virtanen et al. (1988) stated that (racemic) medetomidine up to 30 µM (which would be equivalent to 15 µM dexmedetomidine) failed to affect beating rate or force of contraction in isolated guinea pig atrial preparations but no experimental data were presented to judge these data.

Plasma levels in a clinical trial in human volunteers when dexmedetomidine was given intravenously amounted to about 15 ng/ml (Ebert et al. 2000). Under these conditions, dexmedetomidine halved plasma levels of noradrenaline and adrenaline in these volunteers (Ebert et al. 2000), consistent with stimulation of brain α_2_-adrenoceptors. Low dosages of infused dexmedetomidine reduced mean radial arterial pressure and higher dosage of dexmedetomidine elevated mean radial arterial pressure probably due to peripheral vasoconstriction from stimulation of α_1_-adrenoceptors (Ebert et al. 2000). Dexmedetomidine reduced heart rate by 29 % and cardiac output by 35 % whereas the stroke volume remained unaltered (Ebert et al 2000). Hence, dexmedetomidine does not seem to have a negative inotropic effect but a negative chronotropic effect in patients (Ebert et al 2000).

Plasma concentrations of dexmedetomidine can be doubled when inhibitors of its metabolism are administered (Weerink et al. 2017). Hence, when the degradation of dexmedetomidine is inhibited by other drugs, higher patient plasma concentrations of dexmedetomidine are expected. Whether such elevated concentrations of dexmedetomidine would then be high enough to release noradrenaline in the human heart in patients remains speculative.

### Limitations of the study

One can argue that we have not tested the effects on the sinus node of man directly. We also have not studied human ventricular tissue. The size of the tissue samples was different in mouse and human atrial preparations. In mice, the appendages are less than 2 mm of thickness and their length is about 5 to 8 mm and are only cut once at their edge. The human atrial preparations are cut out of the endocardial tissue. They are of similar size but are cut more often and hence may have more damaged cardiomyocytes and this may be a limitation for direct comparison. Moreover, diffusion limit of oxygen from the buffer should be considered. We have never measured oxygen partial pressure within our multicellular preparations. However, isolated cardiomyocytes would certainly show less propensity for metabolic complications. However, we have failed in the past to isolate in good yield human and mouse atrial cardiomyocytes to obviate possible diffusional limit. However, our contraction studies can usually be performed for at least 2 h before force per se declines. Hence, such diffusion problems should not be a major limit for our findings, but this cannot be completely ruled out. Moreover, it is quite possible that not only species differences but also age differences may exist to explain the divergent effects of dexmedetomidine in mice and men.

In summary, dexmedetomidine is an agonist at human H_2_-histamine receptors in H_2_-TG. In contrast, dexmedetomidine acts as an indirect sympathomimetic agent and an antagonist at H_2_-histamine receptors in HAP, another example of qualitative species differences in cardiac pharmacology.

## Data Availability

All source data for this work (or generated in this study) are available upon reasonable request.
